# Size advantage for male function and size‐dependent sex allocation in *Ambrosia artemisiifolia*, a wind‐pollinated plant

**DOI:** 10.1002/ece3.3722

**Published:** 2017-12-20

**Authors:** Toru Nakahara, Yuya Fukano, Shun K Hirota, Tetsukazu Yahara

**Affiliations:** ^1^ Graduate School of Systems Life Sciences Kyushu University Fukuoka Japan; ^2^ Graduate School of Agricultural and Life Sciences The University of Tokyo Tokyo Japan; ^3^ Institute of Decision Science for a Sustainable Society Kyushu University Fukuoka Japan; ^4^ Department of Biology Faculty of Science Kyushu University Fukuoka Japan; ^5^ Present address: Kawatabi Field Science Center Graduate School of Agricultural Science Tohoku University Miyagi Japan

**Keywords:** *Ambrosia artemisiifolia*, Asteraceae, male fitness, male reproductive investment, paternity analysis, plant height, pollen dispersal, size‐dependent sex allocation, wind‐pollinated plants

## Abstract

In wind‐pollinated plants, male‐biased sex allocation is often positively associated with plant size and height. However, effects of size (biomass or reproductive investment) and height were not separated in most previous studies. Here, using experimental populations of monoecious plants, *Ambrosia altemisiifolia,* we examined (1) how male and female reproductive investments (MRI and FRI) change with biomass and height, (2) how MRI and height affect male reproductive success (MRS) and pollen dispersal, and (3) how height affects seed production. Pollen dispersal kernel and selection gradients on MRS were estimated by 2,102 seeds using six microsatellite markers. First, MRI increased with height, but FRI did not, suggesting that sex allocation is more male‐biased with increasing plant height. On the other hand, both MRI and FRI increased with biomass but often more greatly for FRI, and consequently, sex allocation was often female‐biased with biomass. Second, MRS increased with both height and MRI, the latter having the same or larger effect on MRS. Estimated pollen dispersal kernel was fat‐tailed, with the maximum distance between mates tending to increase with MRI but not with height. Third, the number of seeds did not increase with height. Those findings showed that the male‐biased sex allocation in taller plants of *A. artemisiifolia* is explained by a direct effect of height on MRS.

## 1 INTRODUCTION

Sex allocation in hermaphroditic plants has been a central topic of research in plant ecology since the 1970s. Its theoretical basis was developed by considering resource allocation to male and female functions (Charnov, 1982; Charnov, Bull, & Maynard Smith, 1976), and later extended by Charlesworth and Charlesworth (1981) and Lloyd (1983). Further, Lloyd and Bawa (1984) developed various models applicable to hermaphroditic plants in which gender is continuously adjusted with environmental conditions and argued that size‐dependent sex allocation is not generally supported by their models. Subsequently, however, empirical studies accumulated data showing that size‐dependent gender expression is a common phenomenon in hermaphroditic plants (Klinkhamer, de Jong, & Metz, 1997): Sex allocation is male‐biased in larger plants (i.e., plants with larger reproductive investment or biomass) of wind‐pollinated species, such as *Ambrosia artemisiifolia*,* Xanthium strumarium,* and *Pennisetum typhoides* (Ackerly & Jasieński, 1990; Dajoz & Sandmeier, 1997; Friedman & Barrett, 2011; McKone & Tonkyn, 1986; Solomon, 1989; Traveset, 1992), whereas sex allocation is female‐biased in larger plants of many animal‐pollinated species and some wind‐pollinated species (Bickel & Freeman, 1993; Klinkhamer et al., 1997).

To explain the male‐biased sex allocation in larger plants of wind‐pollinated herbaceous species, the following relationships have been suggested: Male fitness increases linearly with increasing male reproductive investment (MRI) if there is no competition for wind as a pollen vector (Burd & Allen, 1988; Charlesworth & Charlesworth, 1981; Klinkhamer et al., 1997; Sakai & Sakai, 2003); on the other hand, female fitness gain decelerates with increasing female reproductive investment (FRI) if there is local resource competition (LRC) among sib seedlings after germination (De Jong, Van Batenburg, & Van Dijk, 2002; Lloyd, 1982). Linear increase in male fitness gain, with MRI, was recently observed in a wind‐pollinated herb *Beta vulgaris* (De Cauwer, Arnaud, Klein, & Dufay, 2012). However, male fitness gain could be affected not only by MRI but also by plant height, considering that taller wind‐pollinated plants can increase their male fitness gain by releasing pollen from a greater height and dispersing it over greater distances (Burd & Allen, 1988; Klinkhamer et al., 1997; Okubo & Levin, 1989; Sakai & Sakai, 2003; Zhang, 2006). This is because a higher release point allows more horizontal movement, wind velocity increases with increasing elevation both within and above vegetation canopies, and turbulent flow increases at greater height of vegetation (Burd & Allen, 1988; Friedman & Harder, 2005; Levin & Kerster, 1974; Okubo & Levin, 1989). Therefore, taller height may increase fitness gain independently of MRI although taller height also contributes to acquire carbon through a better light condition for photosynthesis (Falster & Westoby, 2003), which is likely to increase reproductive investment. Indeed, in an androdioecious plant *Mercurialis annua* in which erect inflorescence stalks of male plants are located at a taller position than male flowers in leaf axils of hermaphrodites, male plants sired 60% more seeds than hermaphrodites (Eppley & Pannell, 2007). In the study of *M. annua*, however, the effects of height and MRI were also not separated, whereas these two variables are often positively correlated (Weiner & Thomas, 1992). Moreover, in a dioecious plant *Rumex hastatulus* with pollen and seeds dispersed by wind, male plants were taller than female plants at pollen dispersal season although this pattern was reversed at seed maturity (Pickup & Barrett, 2012). However, this study did not examine pollen dispersal and contribution of taller height to male reproductive success (MRS). It remains uncertain whether plant height has significant contribution independently of MRI to pollen dispersal and MRS.

The correlation of height and MRI complicates the theoretical predictions of size‐dependent sex allocation in wind‐pollinated plants (Klinkhamer et al., 1997; Sakai & Sakai, 2003). To resolve this complication, Klinkhamer et al. (1997) distinguished “budget effects” and “direct effects” of plant size on fitness return by defining the former as effects of resource allocation to male and female functions under the resource trade‐off and the latter as a size‐dependent multiplier of male or female fitness function in the fitness formula. Under the direct effects, such as the effects of height on pollen or seed dispersal distance, fitness returns for a given amount of resources invested differ between taller and lower plants, and plants should change gender to their height. On the other hand, if plant size has no direct effects, fitness returns for a given amount of resources invested are equal between larger and smaller plants. In this situation, size‐dependent sex allocation evolves only when at least either of male or female gain curves is nonlinear. Using a different formulation, Sakai and Sakai (2003) distinguished “fecundity effect” and “stature effect” by considering evolutionarily stable sex allocation of large and small plants having different reproductive resources and different pollen and seed dispersal areas in a population. Their model predicts that size‐dependent sex allocation evolves under the relative effects of height on pollen and seed dispersal distance.

**Figure 1:**
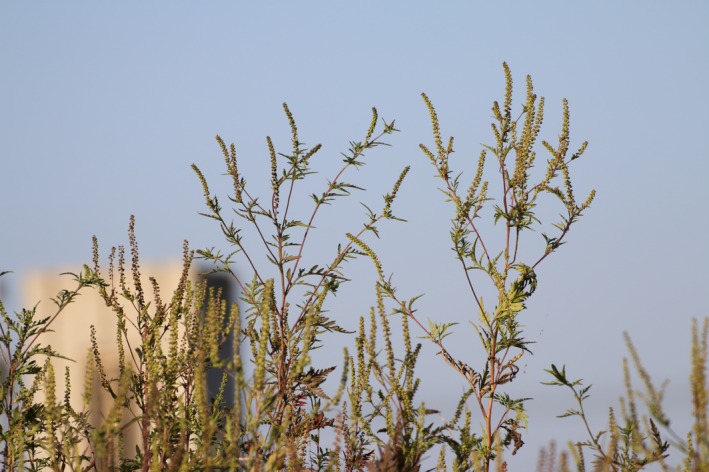
Photograph of *Ambrosia artemisiifolia* taken by Toru Nakahara

These theoretical studies showed the need to quantify the effects of reproductive investment and height separately to better understand the evolution of size‐dependent sex allocation in wind‐pollinated plants. To this end, here we separately quantify the effects of MRI and plant height on MRS and pollen dispersal distance using a wind‐pollinated annual plant, *A. artemisiifolia* (Asteraceae; Figure 1), for the first time. We use the term “size” to describe how large a plant is, measured by parameters such as “biomass” and “reproductive investment,” and the term “height” to describe how tall a plant is. *A. artemisiifolia* is a monoecious species in which MRI (number or weight of male flowers) increases with biomass or height (Friedman & Barrett, 2011; McKone & Tonkyn, 1986; Paquin & Aarssen, 2004) as assessed using univariate analyses. However, previous studies never tried to dissociate the effects of biomass and height on MRI and the effects of MRI and height on MRS in spite that these traits are correlated (Friedman & Barrett, 2011). Thus, we need to examine the independent effects of size and height on sex allocation, MRS, and pollen dispersal distance to evaluate relative contribution of these traits.

In this study, we examined three related questions. First, how do biomass and plant height affect total reproductive investment (TRI), MRI, and FRI? Second, how do MRI and plant height affect MRS and the maximum distance between mates? Third, does plant height affect the number of seeds (i.e., female reproductive success: FRS)? To answer those questions, we conducted two field experiments using artificial populations and estimated the paternity of harvested seeds using six microsatellite markers. We employed a spatially explicit model and multiple regressions for analyzing independent effects of size and height on reproductive success and pollen dispersal distance.

## 2 MATERIALS AND METHODS

### 2.1 Plant species

We used common ragweed *Ambrosia artemisiifolia* L. (Asteraceae), native to North America but spreading as an invasive weed across South America, Europe, Asia, and Australia (Bassett & Crompton, 1975; Essl et al., 2015; Friedman & Barrett, 2011; Shimizu, 2003). It grows in cultivated fields, disturbed habitats, and along the roadside (Bassett & Crompton, 1975). It is a monoecious, wind‐pollinated annual plant with male flowers on racemes at the tips of stems and branches (Payne, 1963). For pollen dispersal, a previous study suggested that relative pollen concentration reduced to 1% at 30–40 m from a pollen source (Dingle, Gill, Wagner, & Hewson, 1959). On the other hand, female flowers are located in small clusters in the axils of leaves and branches (Payne, 1963). Propagation is only by seeds (Friedman & Barrett, 2011) singly contained in achenes having no specialized dispersal morphology and disseminated near the mother plants (Essl et al., 2015). *A. artemisiifolia* produces viable seeds through both self‐ and cross‐fertilization (Bassett & Crompton, 1975), although it is highly outcrossing (Friedman & Barrett, 2008).

### 2.2 Experiment on the effects of height and biomass on reproductive investments

The first experiment was conducted to assess the effects of plant size and height on TRI, MRI, and FRI. For this purpose, we collected seeds from three populations of *A. artemisiifolia* in Japan (Tsukuba City: 36°01′19N, 140°07′08E; Kouka City: 34°55′52N, 136°17′31E; Yamaguchi City: 34°25′59N, 131°46′01E) in 2012. The seeds were stored dry at room temperature and then sown in germination beds (Takii & Co. Ltd., Kyoto, Japan) in March 2013. After germination, seedlings were individually transplanted into plastic flowerpots filled with 3 L of garden soil, and we placed them on a weed‐proof sheet in the experimental field of Kyushu University. The potted plants were randomly spaced 50 cm apart. They were watered daily, and their positions were randomly changed once in each month until harvest. We occasionally sprayed 0.1% v/v fenitrothion to control herbivorous insects. We recorded final plant heights (cm) at seed maturity. Seed maturity was visually evaluated by the size and color of seeds. After harvesting the plants and drying them in the shade, we measured the weights of above‐ground components, male flowers, and seeds as indicators of biomass, MRI, and FRI, respectively. We did not weigh below‐ground components following some previous studies (Ackerly & Jasieński, 1990; Friedman & Barrett, 2011). We excluded plants that dropped more than 25% of male flowers before harvest. As a result, we used 22 of 60, 16 of 20, and 17 of 20 plants from the plant populations of Tsukuba, Kouka, and Yamaguchi, respectively, for the analysis of TRI, MRI, and FRI.

### 2.3 Experiment on the effects of height and MRI on MRS and pollen dispersal

**Figure 2:**
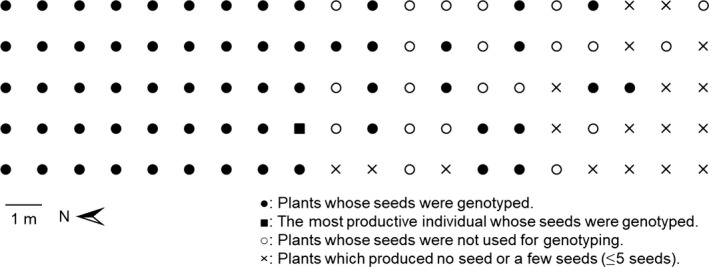
Plant distribution in the field on the experiment for evaluating effects of MRI and height on MRS

The second experiment aimed at assessing the effects of size and height on fitness components and pollen dispersal. Potted plants used in the experiment were grown from seeds collected in Tsukuba, Japan, in 2011. We randomly arranged 100 potted plants before flower bud formation in a square 5 × 20 reticular pattern with a distance of 1 m between pots (4 m east–west; 19 m north–south) on a weed‐proof sheet in the experimental fields of Kyushu University in 2012 (Figure 2). These plants were selected to allow assignment of 80% seeds to their fathers under the 95% confidence level from 320 plants genotyped with 10 microsatellite markers using CERVUS v. 3.0.7 (Appendix S1, Genton et al., 2005; Abercrombie et al., 2009; Marshall, Slate, Kruuk, & Pemberton, 1998). We set this percentage of assignment to allow pollination from unknown populations that could be in the vicinity of the experimental population, although the closest known naturalized population was about 7.5 km away. We applied water to plants daily until harvest. Periodically, we sprayed 0.1% w/v fenitrothion to prevent insect infestation. About 7 months after the first germination, the plants stopped growing and we recorded their height to the nearest 0.1 cm and the total length of all racemes to the nearest 1 mm. Raceme length was considered as a good indicator of MRI because it was strongly correlated with the number of male flowers (*r *=* *.788, *p *<* *.001, *N* = 55, apical raceme length vs. number of male flowers) and the weight of male flowers (*r *=* *.711, *p *<* *.001, *N* = 55, apical raceme length vs. weight of male flowers) as observed in a preparatory experiment (details not shown). At maturity, we harvested seeds in the field and counted them in the laboratory, but we failed to record total raceme length on two plants. Plants from the 11 southern rows had high maleness, including 15 plants that produced no or few female flowers. The southern rows were near a road, which may cause negative effects due to the roadside environment, but the reason for the high maleness remains uncertain. One plant designated as “the most productive individual” produced 862 seeds, and the others designated as “less productive individuals” produced 157.3 ± 158.9 seeds (mean ± *SD*) per plant.

### 2.4 Polymerase chain reaction (PCR) analysis of seeds

PCR analysis was conducted following Hirota et al. (2013), with modifications described below. We selected 2,326 seeds from 12,453 seeds produced by 45 genotyped individuals from the nine northern rows: We randomly selected 226 seeds from the most productive individual and 47.8 ± 5.63 seeds per plant (mean ± *SD*) from 44 less productive individuals to estimate their paternity and the distances between outcrossing mates. The percentage of examined seeds from the most productive individuals was 26.2%, which was almost same as the mean value of less productive individuals (26.2%). Of 3,988 seeds produced by other 55 genotyped individuals from the 11 southern rows, we selected 408 seeds from 16 individuals: We randomly selected 48 seeds from an individual and 24 seeds per plant from the other 15 individuals which produced over 40 seeds. Seeds were soaked overnight in distilled water, and their testae were peeled off using tweezers. Each seed was placed in a 96‐well FrameStar plate (4titude, Surrey, UK) containing extraction buffer (0.01% SDS; 0.02% Proteinase K (Wako, Osaka, Japan); 0.01 mol/L Tris‐HCl, pH 7.8; 0.01 mol/L EDTA), with one seed per well. After crushing each seed using a 10 μl pipette tip, the plate was incubated for 180 min at 65°C and heated for 10 min at 95°C. The extracted DNA solution was diluted fourfold with distilled water, distributed to separate plates in two wells, and then used as a PCR template. PCR was conducted on a TaKaRa PCR Thermal Cycler Dice Gradient (TaKaRa, Shiga, Japan). Forward primers were labeled with a fluorescent dye (G5 dye set: 6‐FAM, VIC, NED, or PET; Applied Biosystems, Foster City, CA, USA) simultaneously for five of 10 microsatellite loci. Multiplex PCR amplification was conducted using the Multiplex PCR Kit (Qiagen K.K., Tokyo, Japan) in a 6 μl reaction consisting of 1 ×  Qiagen Multiplex PCR Master Mix, 0.2 μmol/L each primer, and 1 μl of a template extract from a seed. The PCR conditions were as follows: 95°C for 5 min (hot start); 28 cycles at 95°C for 30 s, 57°C for 90 s, and 72°C for 30 s; and a final extension step at 60°C for 30 min. PCR products were separated by electrophoresis on an ABI 3730 DNA Analyzer (Applied Biosystems), and allele sizes were determined using the Gene Mapper 4.1 fragment analysis software (Applied Biosystems). The above procedure was repeated for another plate on which microsatellites of the remaining five loci were amplified. Samples in which no locus was amplified were removed from subsequent analyses.

### 2.5 Paternity analysis for estimating selection gradients on MRS and pollen dispersal kernel in neighborhood model

Before paternity analysis, we checked null allele frequency using INEST 2.1 (Chybicki & Burczyk, 2009), and we used six of 10 loci in which null allele frequency was <0.34 in seed samples. We selected sets of seeds and parents genotyped at least for five of the six loci. For paternity analysis, we employed the program NM+ v. 1.1 (Chybicki & Burczyk, 2010, 2012) instead of MEMM (Klein, Desassis, & Oddou‐Muratorio, 2008) because the former enables us to estimate not only pollen dispersal kernel but also selection gradients on MRS in which we can evaluate relative contribution of height and MRI; we applied a neighborhood model (Burczyk, Adams, Moran, & Griffin, 2002) implemented in the program NM+ to 2,102 seeds which were genotyped. On the other hand, NM+ does not estimate individual reproductive success and we need to examine how it could vary with the effects of the spatial position of individual plants in the experimental population. Using NM+, we can consider the spatial effects by estimating selection gradients and pollen dispersal kernel with different neighborhood radii and compare the results. Here, we assumed neighborhood radii as 2, 5, 10, or 15 m, a typing error rate as constant (0.01) and the precision of convergence (“stop” criterion) as 0.001. We used the following exponential power function for estimating pollen dispersal kernel (Austerlitz et al., 2004):

$$P\left(r\right)\;=\;\frac{b}{2\pi a^{2}\Gamma \left(\frac{2}{b}\right)}\text{exp}\left[-{\left(\frac{r}{a}\right)}^{b}\right]$$

where *r* is pollination distance, Г is the gamma function, and *a* and *b* are scale and shape parameters of dispersal kernels, respectively. When *b *=* *1, kernel follows a simple exponential distribution. On the other hand, when *b *<* *1 or *b *>* *1, kernel is fat‐tailed or thin‐tailed, respectively (Austerlitz et al., 2004). We estimated the following parameters using the Newton–Raphson algorithm: pollen immigration rate (*m*_*p*_), scale and shape parameters of dispersal kernels (*a* and *b*_*p*_, respectively), mean pollen dispersal distance (*d*_*p*_), directionality effect (*K*_*p*_), the prevailing direction of dispersal (θ_*p*_), and selfing rate (*s*). We also estimated selection gradients β_1_ for height, β_2_ for total raceme length, and β_3_ for total number of seeds of neighboring individuals on MRS using a function of the program (Chybicki & Burczyk, 2010, 2012), in which the equation was represented as follows:

$$\varphi_{i}\;=\;\text{exp}\left(\beta_{1}x_{i1}\;+\;\beta_{2}x_{i2}\;+\;\beta_{3}x_{i3}\right)$$

where $\varphi_{i}$ is MRS of *i*th individual; *x*_*i*1_, *x*_*i*2_, and *x*_*i*3_ are standardized height, total raceme length, and total number of seeds of neighboring individuals of *i*th individual, respectively. Significance of selection gradients and relative effect size was examined based on a 95% confidence interval calculated from standard errors of selection gradients assuming normal distribution.

### 2.6 Paternity analysis for evaluation of effects of MRI and height on pollen dispersal distance

To evaluate effects of MRI and height on pollen dispersal distance, we conducted paternity analysis also using CERVUS because the NM+ program did not output the paternity of each seed. We selected sets of seeds and parents genotyped at least for five of the six loci. We computed the maximum‐likelihood paternity of each seed using the multilocus genotypes of candidate paternal plants. For each seed tested, the paternity likelihood of each candidate father was examined by a LOD score, the ratio of probabilities calculated based on the multilocus genotypes of the tested seed, a maternal parent, and candidate paternal plants, and allele frequencies of loci used (Meagher, 1986). When the significance of the paternity determination was <95%, those seed samples were excluded from subsequent analyses. We analyzed data by allowing partial selfing, because *A. artemisiifolia* is mostly outcrossing but self‐compatible (Bassett & Crompton, 1975) and our allele frequency analysis in seed populations showed significant deficiency of heterozygosity (Appendix S2). By this analysis, we estimated the paternity of 1,306 seeds and then determined maximum distances between mates.

### 2.7 Statistical analysis

For all statistical analyses, we used the R software, v. 3.1.1 (R Core Team, 2014). Whether plants with larger biomass and taller height allocate more resources to total reproduction, male and female functions were tested using ANCOVA in which TRI (i.e., the sum of total weight of male flowers and seeds), MRI, and FRI (i.e., total weight of male flowers or seeds) were explained by a multiple linear regression model of above‐ground dry biomass and height. Plant height and biomass were moderately correlated (*r *=* *.49, *p *<* *.001, details not shown). *p‐*Values were calculated using the *F* test in all regression analyses except where stated. To compare relative contributions of height and dry biomass, both variables were standardized (mean* *=* *0, *SD *=* *1) and then included as explanatory variables. We also included population origin and the interaction between a size (dry biomass) or height and population as explanatory variables. Anywhere there was significant interaction, we further tested the significance of simple main effects by post hoc tests using the Bonferroni's adjustment. If there was no significant interaction, it was removed from explanatory variables.

We also conducted the regression analyses using measurements not standardized. MRI and FRI calculated from the obtained regression equations, represented as *f*_*M*_ and *f*_*F*_ *,* respectively, were used for describing the relationship between sex allocation and size (dry biomass) or height as *f*_*M*_/(*f*_*M*_ **+ *f*_*F*_).

Whether larger MRI and/or taller height cause longer pollen dispersal distance was tested using ANOVA, where the maximum distance between maternal and paternal plants was explained by a multiple linear regression model of total raceme length and height. The maximum distance was chosen as an indicator of pollen dispersal distance because it is more sensitive to the tail of pollen dispersal kernel. The number of neighboring individuals was also included as a covariate to consider an edge effect. Moreover, considering the rectangular shape of the experimental array, the mean distance to the other individuals whose seeds were genotyped was included in the model as another covariate; this mean distance is larger in individuals locating in more peripheral positions in the experimental population (Figure 2).

Whether plants with taller height produced more seeds was tested using a GLM in which seed count was explained by a multiple linear regression model of height with a log link function assuming that errors follow a quasi‐Poisson distribution. The number of neighboring individuals and an effect of total male fecundity (total raceme length) of neighboring individuals were considered as covariates. Among 100 individuals, 13 plants surrounding two plants for which we failed to measure total raceme length (see above) were excluded from this analysis. Plants which produced no or a few seeds (≤5 seeds) on the southern 11 rows were also removed. Variables were standardized (mean* *=* *0, *SD *=* *1). Quadratic terms and interactions between these variables were not included in the model because those were not significant.

The variance inflation factor of the explanatory variables was calculated to check for multicollinearity between variables in all regression analyses. We considered that there was no multicollinearity as the variance inflation factor was <10 (Hair, Anderson, Tatham, & Black, 1998).

## 3 RESULTS

### 3.1 The relationship between TRI and biomass or height

TRI was not significantly associated with height, whereas the effect of dry biomass on TRI was significant and varied according to the population of origin (Table 1; Appendix S3). In post hoc tests with Bonferroni's adjustment to examine the main effect of dry biomass in each population, TRI was positively associated with dry biomass in the populations of Tsukuba (Appendix S3A; estimate ± *SE *=* *0.973 ± 0.163, *F*_1,19_ **=* *35.487, *p *<* *.001) and Kouka (Appendix S3B; estimate ± *SE *=* *0.942 ± 0.148, *F*_1,13_ **=* *40.410, *p *<* *.001), but not in Yamaguchi (Appendix S3C; estimate ± *SE *=* *0.371 ± 0.160, *F*_1,14_ **=* *5.366, *p *=* *.109).

**Table 1:** Effects of plant height, dry biomass, and population origin on total, male and female reproductive investments in *Ambrosia artemisiifolia*. Plant height and dry biomass were standardized

	Effects on total reproductive investment				Effects on total weight of male flowers				Effects on total weight of seeds			
	Estimate	*SE*	*F*	*p*	Estimate	*SE*	*F*	*p*	Estimate	*SE*	*F*	*p*
(Intercept)	3.753	0.143	–	–	1.110	0.066	–	–	2.646	0.119	–	–
Height	−0.072	0.118	*F*_1,48_ * *=* *0.373	.544	0.137	0.057	*F*_1,50_ * *=* *5.789	.020	−0.210	0.098	*F*_1,48_ * *=* *4.629	.036
Dry biomass	1.328	0.185	*F*_1,48_ * *=* *58.444	<.001	0.127	0.048	*F*_1,50_ * *=* *7.095	.010	1.215	0.153	*F*_1,48_ * *=* *58.567	<.001
Population			*F*_2,48_ * *=* *5.583	.007			*F*_2,50_ * *=* *4.096	.023			*F*_2,48_ * *=* *6.061	.004
Tsukuba vs. Kouka	−0.425	0.218			−0.224	0.115			−0.579	0.181		
Tsukuba vs. Yamaguchi	−0.652	0.244			0.154	0.102			−0.420	0.202		
Dry biomass × population			*F*_2,48_ * *=* *13.100	<.001	–	–	–	–			*F*_2,48_ * *=* *20.528	<.001
Tsukuba vs. Kouka	−0.278	0.251							−0.269	0.208		
Tsukuba vs. Yamaguchi	−1.061	0.226							−1.096	0.188		

### 3.2 The relationship between MRI or FRI and biomass or height

The ratio of MRI (the weight of male flowers divided by the total weight of male flowers and seeds) was 0.34 ± 0.10 (mean ± *SD*). Height‐ and biomass‐dependent increase in MRI was found in all three populations: the total weight of male flowers was significantly positively associated with height, dry biomass, and population origin (Table 1). On the other hand, height‐dependent decrease and biomass‐dependent increase in FRI was found in three and two populations, respectively: The total weight of seeds was negatively associated with height, and the interaction between dry biomass and population origin was significant (Table 1). As such, we examined the main effect of dry biomass in each population by post hoc tests with Bonferroni's adjustment (Appendix S3A–C). The total weight of seeds was significantly positively associated with dry biomass in Tsukuba (estimate ± *SE *=* *0.899 ± 0.125, *F*_1,19_ **=* *51.834, *p *<* *.001) and Kouka (estimate ± *SE *=* *0.811 ± 0.117, *F*_1,13_ **=* *48.183, *p *<* *.001), but not in Yamaguchi (estimate ± *SE *=* *0.196 ± 0.152, *F*_1,14_ **=* *1.673, *p *>* *.05).

The relative contribution of height and dry biomass to MRI and FRI was tested by comparing standardized partial regression coefficients. For MRI, the regression coefficients showed no significant difference (*F*_1,50_ **=* *0.012, *p *=* *.915; Table 1). On the other hand, for FRI in each population, the coefficient of dry biomass was significantly larger than that of height in Tsukuba (*F*_1,19_ **=* *28.008, *p *<* *.001, Appendix S3A) and Kouka (*F*_1,13_ **=* *16.413, *p *=* *.001, Appendix S3B), but the difference was marginal in Yamaguchi (*F*_1,14_ **=* *4.215, *p *=* *.059, Appendix S3C).

**Figure 3:**
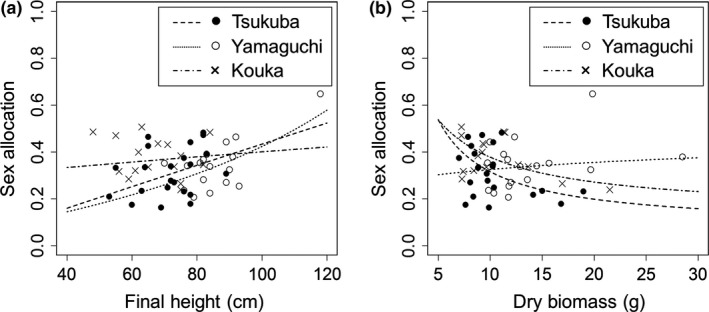
Relationship between a size trait (a: height or b: biomass) and sex allocation. Sex allocation was defined as total weight of male flowers divided by total weight of male flowers and seeds. Each curve is drawn using linear regression equations of total weight of male flowers and seeds to biomass and height (not standardized), namely *f*_*M*_/(*f*_*M*_ **+ *f*_*F*_)

The relationship between sex allocation (proportion of MRI) and plant height or dry biomass (Figure 3) was overlaid with curves derived from multiple regression analyses for MRI and FRI, namely *f*_*M*_/(*f*_*M*_ **+ *f*_*F*_). Male sex allocation increased with height in all three populations (Figure 3a) but did not increase with biomass in Tsukuba and Kouka populations where total weight of seeds significantly increased with biomass (Figure 3b).

### 3.3 The relationship between MRS and MRI or height

**Figure 4:**
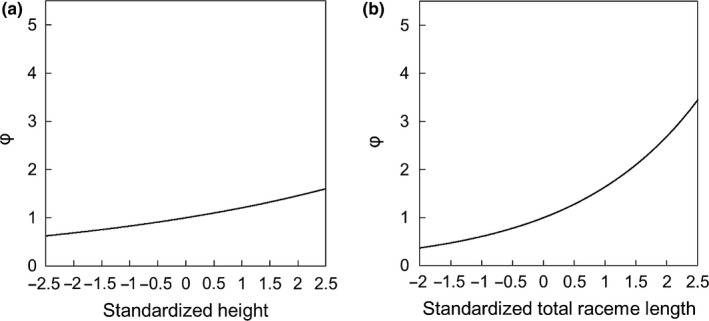
Relationship between (a) height or (b) total raceme length and MRS (ψ). The lines were derived from an equation of selection gradient analysis using NM+ program when neighborhood size was 2 m radius (Table 2)

In the selection gradient analysis by NM+ program, irrespective of neighborhood radius, MRS significantly increased with height (β_1_: 0.120–0.188; Table 2; Figure 4a) and total raceme length (β_2_: 0.484–0.518; Table 2; Figure 4b) because 95% confidence intervals of β_1_ and β_2_ were larger than zero. When neighborhood radius was set as 5, 10, and 15 m, β_2_ was significantly larger β_1_ because 95% confidence intervals of the difference of selection gradients (β_2_ − β_1_) were larger than zero. When neighborhood radius was set as 2 m, there was no significant difference between β_1_ and β_2_.

**Table 2:** Parameters of pollen dispersal estimated using NM+ program. Parentheses represent standard errors

Neighborhood size (radius)	Parameters^a^							Selection gradients^b^		
	*m*_*p*_	*s*	*d*_*p*_	*b*_*p*_	*K*_*p*_	θ_*p*_	*a*	β_1_	β_2_	β_3_
2 m	0.650	0.047 (0.005)	13.641 (12.704)	0.170 (0.103)	0.186 (0.062)	2.414 (21.124)	2.32 × 10^−6^	0.188 (0.045)	0.495 (0.056)	0.070 (0.076)
5 m	0.457	0.049 (0.005)	15.042 (4.796)	0.163 (0.039)	0.216 (0.053)	26.037 (14.898)	8.55 × 10^−7^	0.120 (0.034)	0.518 (0.042)	0.120 (0.055)
10 m	0.397	0.048 (0.005)	23.687 (4.599)	0.159 (0.024)	0.246 (0.050)	21.505 (12.970)	9.26 × 10^−7^	0.139 (0.032)	0.496 (0.040)	0.106 (0.050)
15 m	0.386	0.048 (0.005)	39.817 (8.369)	0.138 (0.020)	0.246 (0.050)	21.796 (12.924)	4.27 × 10^−8^	0.143 (0.032)	0.484 (0.039)	0.090 (0.049)

a *m*_*p*_: pollen immigration rate, *s*: selfing rate, *d*_*p*_: mean pollen dispersal distance, *b*_*p*_: shape parameter of a dispersal kernel, *K*_*p*_: directionality effect, θ_*p*_: the prevailing direction of dispersal, *a*: scale parameter of a dispersal kernel.

b Selection gradients β_1_ for height, β_2_ for total raceme length, and β_3_ for total number of seeds of neighboring individuals on MRS.

### 3.4 The relationship between pollen dispersal ability and MRI or height

**Figure 5:**
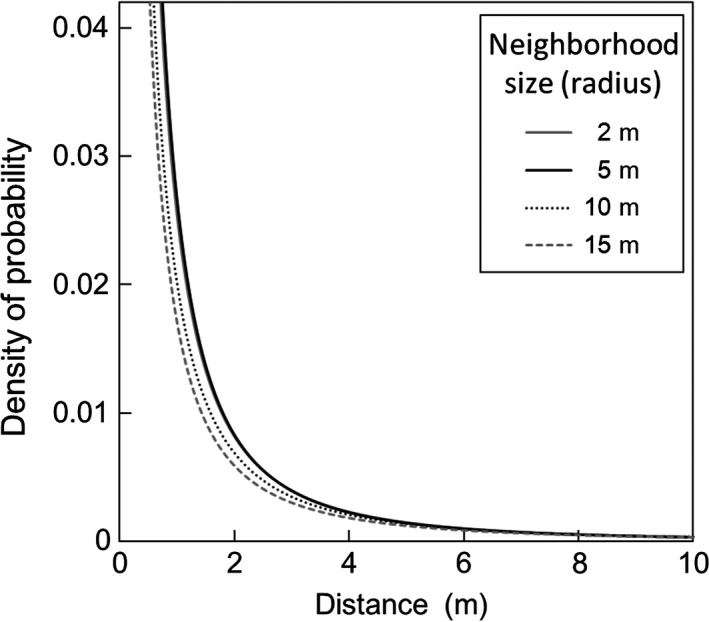
Pollen dispersal kernel estimated using NM+ program

The longest mating distance observed between two parents was 18.0 m, close to 19.42 m, the longest distance between two individuals in the field. In pollen dispersal kernel estimated by NM+ program, the shape parameter *b*_*p*_ was below unity, indicating fat‐tailed distribution (Table 2; Figure 5).

The effect of MRI or height on pollen dispersal distance was analyzed by the maximum distance to mates. The distance did not significantly change with height but significantly increased with total raceme length. It was also positively associated with the number of neighbor individuals and the mean distance to the other individuals (Table 3).

**Table 3:** Effects of plant height and total raceme length on the maximum distance between mates in *Ambrosia artemisiifolia*. These effects were estimated using a generalized linear model

	Estimate	*SE*	*F*_1,80_	*p*
(Intercept)	−6.733	2.972	–	–
Height (cm)	0.038	0.027	2.068	.154
Total raceme length (mm)	0.001	0.0003	4.739	.032
Number of neighbor individuals	0.502	0.198	6.417	.013
Mean distance to detectable candidate mothers	1.300	0.150	74.865	<.001

### 3.5 The relationship between seed production and plant height

**Figure 6:**
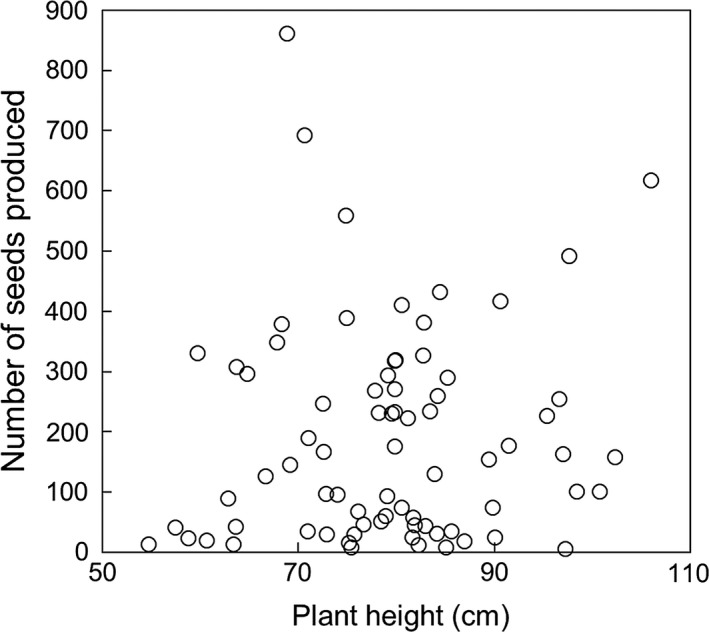
The relationship between plant height and number of seeds. Plants which we did not use for a GLM analysis were removed

In our analysis using 75 of 100 individuals, the number of seeds produced was not significantly associated with height. On the other hand, it was positively associated with the total raceme length of neighboring individuals and negatively associated with the number of neighbor individuals (Table 4; Figure 6).

**Table 4:** Effects of plant height, the number, and male fecundity of neighboring individuals on the number of seeds in *Ambrosia artemisiifolia*. Explanatory variables were standardized

	Estimate	*SE*	*F*_1,71_	*p*
(Intercept)	5.124	0.116	–	–
Height	0.016	0.102	0.024	.876
Number of neighbor individuals	−0.497	0.137	14.197	<.001
Total raceme length of neighboring individuals	0.572	0.140	18.318	<.001

## 4 DISCUSSION

Our study provided quantitative evidence for the sex allocation change with size and height in *A. artemisiifolia* and the independent effects of reproductive investment and height on MRS and pollen dispersal distance; neighborhood radii of 2, 5, 10, and 15 m gave almost identical results (Figure 5; Table 2) and the effects of spatial geometry in the experimental population are considered negligible. First, biomass had positive effects on both MRI and FRI, except for one population, but height had a positive effect only on MRI (Table 1; Appendix S3). Second, MRS significantly increased with MRI and height, although the effect of plant height was equal to or smaller than the effect of MRI (Table 2; Figure 4). Third, maximum distance between mates increased with MRI but did not change with height (Table 3). Forth, seed production was not significantly associated with plant height (Table 4; Figure 6). We discuss these findings in detail below and consider its implication on the evolution of sex allocation.

### 4.1 Effects of plant size and height on TRI, MRI, and FRI

Theoretical models assumed the size‐dependent increase in reproductive resource allocation in plants when considering the evolution of size‐dependent sex allocation (Klinkhamer et al., 1997; Sakai & Sakai, 2003). Our findings in the analysis of TRI are consistent with this assumption (Appendix S3).

In theoretical models, the effects of reproductive resource and plant height are not always formulated as easily measurable ways, although Klinkhamer et al. (1997) distinguished “budget effect” and “direct effect,” and Sakai and Sakai (2003) distinguished “fecundity effect” and “stature effect.” Here, we empirically showed that not FRI but MRI increased with height (Table 1), indicating that sex allocation is more male‐biased in taller plants (Figure 3a). In *A. artemisiifolia*, greater MRI relative to FRI with increasing plant height has been reported previously (Ackerly & Jasieński, 1990; Friedman & Barrett, 2011; McKone & Tonkyn, 1986; Paquin & Aarssen, 2004; Traveset, 1992).

While sex allocation was more male‐biased with height in all three populations, it was more female‐biased with biomass in two populations (Figure 3b). In the population of Yamaguchi, sex allocation ratios seem to increase (i.e., changed toward more male‐biased) with biomass (Figure 3b), but the reproductive investment‐biomass regression was significant only for male, and thus, more male‐biased sex allocation is only weakly suggested. In a previous study using *A. artemisiifolia*, Friedman and Barrett (2011) showed that the numbers of male and female flowers varied among families under equivalent light conditions or between different light conditions. This shows that both genetic and environmental factors affect male and female reproductive allocations (Friedman & Barrett, 2011). As our experiment was conducted under similar conditions, our results suggest that the differences in the trend of sex allocation with biomass between plant populations were caused partly by the genetic variation between those populations.

### 4.2 Effects of plant size and height on MRS and pollen dispersal distance

Sex allocation in wind‐pollinated plants is likely to have evolved under the effects of reproductive investment and height (Klinkhamer et al., 1997). In *A. artemisiifolia,* both MRI and height had significant positive effects on MRS, although the height effect was smaller than the effect of MRI when large neighborhood radius was assumed (Table 2; Figure 4). These findings agree with the argument of Klinkhamer et al. (1997) that plant size can influence fitness returns in two different ways.

The effect of MRI or height on MRS has been studied in wind‐pollinated plants, including a perennial herb *B. vulgaris* (De Cauwer et al., 2012) and some conifers (Burczyk, Adams, & Shimizu, 1996; Burczyk & Prat, 1997; Schoen & Stewart, 1986; Torimaru, Wennstrom, Lindgren, & Wang, 2012), but those studies considered only either MRI or height. In contrast, we assessed the effects of these two components on MRS simultaneously and showed that MRS tends to increase with MRI and height.

For pollen dispersal, the estimated kernel was fat‐tailed, indicating that the long‐range decay of dispersal probability is slower than the expectation from the exponential distribution (Austerlitz et al., 2004; Figure 5). This finding agrees with a general pattern of pollen dispersal in wind‐pollinated plants (Gerber et al., 2014; Levin & Kerster, 1974; Oddou‐Muratorio, Klein, & Austerlitz, 2005). This fat‐tailed distribution may be due to the position effect that a higher release point allows more horizontal movement and also the aerodynamic property that wind speed and turbulent flow are greater at a higher position where horizontal movement of pollen is favored (Burd & Allen, 1988). Under this fat‐tailed distribution, it is expected that taller plants have candidate mates across a large pollen dispersal area (Burd & Allen, 1988). However, the maximum distance between mates was not significantly associated with plant height (Table 3). This result may be due to an underestimation of the maximum distance because the mean pollen dispersal distance (*d*_*p*_) was 13.6–39.8 m, whereas the maximum distance was 19.42 m. A positive effect of height on the maximum distance could be detected if we use larger experimental populations. Moreover, high null allele frequencies (Appendix S2) may cause underestimation of the maximum pollen dispersal distance.

### 4.3 Effects of plant height on seed production

Seed production was significantly associated with male fecundity around the plant and the number of surrounding individuals, but not with plant height (Table 4; Figure 6) indicating that *A. artemisiifolia* produces a fixed number of seeds regardless of its height. In plants, there is a general allometric relationship that seed production increases with biomass (Weiner, Campbell, Pino, & Echarte, 2009) and that biomass positively correlates with plant height (Weiner & Thomas, 1992). Thus, it may be expected that seed production increases with height. However, in *A. artemisiifolia*, there was a weak or moderate correlation between plant height and biomass (Friedman & Barrett, 2011; this study).

This weak or moderate correlation between plant height and biomass may explain the decoupled relationship between seed production and height in *A. artemisiifolia*. As *A. artemisiifolia* is neither anemochorous nor zoochorous, most seeds are disseminated near the mother plants (Essl et al., 2015), suggesting that height‐dependent seed dispersal is unlikely. Therefore, excessive seed production may increase the risk of LRC among sib seedlings (De Jong & Klinkhamer, 1994; Lloyd, 1982). Under this relationship, fixed seed production regardless of its height as observed in the present study is expected to be advantageous. On the other hand, Paquin and Aarssen (2004) reported that the number of seeds in *A. artemisiifolia* increased with plant height when the variation in plant height was induced by varying light and nutrient conditions. This plasticity of seed production with height may be advantageous if the risk of LRC varies with light and nutrient environments.

Previous studies assumed that female fitness gain is expected to level off with plant size under increasing LRC among seedlings derived from larger plants (De Jong & Klinkhamer, 1994; Klinkhamer et al., 1997). We did not test this assumption because we did not examine LRC. It is needed to examine how the survivorship of seedlings under LRC changes with resource allocation and seed dispersal distance.

### 4.4 Why sex allocation is more male‐biased in taller plants?

We found that reproductive resource allocation to male function increased with plant height in all populations. This finding may support the prediction of Klinkhamer et al. (1997) that most wind‐pollinated species should adjust their gender according to height because the direct effects of height are generally expected in wind pollination. If there is no direct effect, the theory predicts that sex allocation depends on budget invested in reproduction when at least one of the fitness gain curves is nonlinear and more specifically it is male‐biased when MRS increases with reproductive biomass investment but FRS saturates by LRC. However, this prediction was not always supported in *A. artemisiifolia* because sex allocation was more male‐biased with biomass only in one of three populations. On the other hand, sex allocation was more male‐biased with height in all three populations*,* suggesting the presence of direct height effects on MRS. Klinkhamer et al. (1997) also predicted that fitness returns per absolute investment differ between small and large plants under the direct effects but those are equal between different size classes if there is no direct effect. The relationships between male fitness returns (MRS) and absolute investment (MRI adjusted for height) were tested in the analysis using NM+ in which the spatially explicit model is implemented, selection gradients were significant for both height and MRI, supporting the presence of the direct effect of height.

On the other hand, Sakai and Sakai (2003) argued that the positive height effect on pollen dispersal distance and MRS is not sufficient to explain the more male‐biased sex allocation in taller plants, because height can also change seed dispersal distance, and the relative effects of height on pollen and seed dispersal distance change with size structure of a population. According to their game model considering a population composed of “large” and “small” plants, large plants tend to be more male‐biased or more female‐biased if *t *> *k* or *t *< *k*, respectively, where *t* is relative fecundity (fecundity of “large” plants divided by fecundity of “small” plants) and *k* is relative size of seed dispersal area of the large plants to the small plants.

Our results indicated that *t* is larger than 1 because TRI was significantly larger in plants with larger biomass than in smaller plants, and we suggest that *k* is approximately unity because seed dispersal distance is unlikely dependent of plant height in *A. artemisiifolia*. Thus, the condition *t *> *k* required for the evolution of male‐biased evolutionarily stable sex allocation in large plants (Sakai & Sakai, 2003) may be satisfied in *A. artemisiifolia*. However, the model of Sakai and Sakai (2003) does not consider the shape of pollen dispersal kernel, which is likely linked to the relative size of pollen dispersal area (*l*) of the large plants to the small plants. If fat‐tailed pollen dispersal is assumed in the model, the class of plants that is male‐biased may be determined not only by *k* and *t* but also by *l,* as suggested in many previous studies (Burd & Allen, 1988; Friedman & Barrett, 2009; Klinkhamer et al., 1997; Zhang, 2006). Further theoretical studies are needed to better understand this hypothesis.

## 5 CONCLUSION

In *A. artemisiifolia*, not biomass but height is a primary determinant of male‐biased sex allocation. On the other hand, both MRI and height are significant determinants of MRS. This is the first documentation of evidence from multivariate analyses for the classic idea that taller wind‐pollinated plants can increase their male fitness gain (Burd & Allen, 1988; Klinkhamer et al., 1997; Okubo & Levin, 1989; Sakai & Sakai, 2003; Zhang, 2006). To generalize our conclusion, further studies using the multivariate analyses are needed in which we recommend to use larger sample size because the selection gradient of MRI is likely to be much larger than that of height. Further studies are also needed for natural populations where environmental conditions and relationships with other species may have significant effects on MRS. Our results suggest that the relationship between sex allocation and biomass may vary genetically among natural populations but why it varies remain to be explained. As suggested by Sakai and Sakai (2003), sex allocation in wind‐pollinated plants may have evolved under more complicated mechanisms than previously thought, and our study showed that the evaluation of independent effects of biomass and height is crucial to resolve this complication.

## CONFLICT OF INTEREST

None declared.

## AUTHOR CONTRIBUTION

TN, YF, SKH, and TY conceived and designed the study. TN and YF collected the data and TN analyzed it. TN wrote the manuscript, with the contribution of all authors.
