# Supplementary material for: Size advantage for male function and size‐dependent sex allocation in Ambrosia artemisiifolia, a wind‐pollinated plant
Source: Ecol Evol. 2017 Dec 20;8(2):1159–70. doi: 10.1002/ece3.3722 (PMC5773293; doi:10.1002/ece3.3722)
Supplement: Supplementary file 1 [file ECE3-8-1159-s001.docx]

**Appendix S1. Microsatellite markers used in genotyping.** Pigtails (GTTTCTT) were added to the reverse primers to facilitate accurate genotyping [1*]. Six of 10 markers (*Amb82*, *Ambart09*, *Ambart21*, *Ambart18*, *Ambart06* and *Ambart17*) were used for paternity analysis.

| Locus | Repeat Motif | Primer sequences (5′→3′) | Reference |
| --- | --- | --- | --- |
| *Ambart04* | (ATAC)_22_ | F: AGGGGTTAGTTCTCTTAGCATCC | [2†] |
|  |  | R: TCCGCGTGATTGAGTTTATG |  |
| *Ambart06* | (TC)_16_(AC)_9_ | F: AAAGTGCCACAAACACCTTG | [2] |
|  |  | R: CTGCCAAAACAACTGGAAGG |  |
| *Ambart09* | (AC)_13_ | F: TGCTGCATAAATTGCTCCAC | [2] |
|  |  | R: GTATTCGAGCCGAGCATACC |  |
| *Ambart17* | (GTAT)_13_ | F: CCATGGTAATACTATCCTTACAACAC | [2] |
|  |  | R: TCAAGGACCACTGGGACATAC |  |
| *Ambart18* | (AG)_18_ | F: TGGTGGTGGAGCTTTTTGAC | [2] |
|  |  | R: AGCACCTCACGTTCCTCTTC |  |
| *Ambart21* | (CT)_15_ | F: TCACAGGCATAATTCACTTGG | [2] |
|  |  | R: TCTATGTGGTTCTCATAATTCTTCA |  |
| *Ambart24* | (GA)_14_ | F: GATCATTGCTGCAGTTTCAAC | [2] |
|  |  | R: CATCATTCAAATTTTCCATATTGC |  |
| *Amb12* | (AC)_6_ | F: GACGAAAGCAACCAAACACA | [3‡] |
|  |  | R: GGAAAAATGCATGCACAAAA |  |
| *Amb30* | (AC)_9_ | F: AGAGATGAAATAACGAGCAACAAA | [3] |
|  |  | R: AATGAGGATGCATTGGGTTT |  |
| *Amb82* | (AG)_22_ | F: AAACAACTAGTGTGTGTTTCAGTGTG | [3] |
|  |  | R: GTCTTCGGCCGTAAAATGAC |  |

*1. Brownstein, M.J., Carpten, J.D. & Smith, J.R. 1996. Modulation of non-templated nucleotide addition by Taq DNA polymerase: primer modifications that facilitate genotyping. *BioTechniques* **20:** 1004-1006, 1008-1010.

†2. Abercrombie, L.G., Anderson, C.M., Baldwin, B.G., Bang, I.C., Beldade, R., Bernardi, G. *et al.* 2009. Permanent genetic resources added to molecular ecology resources database 1 January 2009–30 April 2009. *Mol. Ecol. Resour.* **9:** 1375-1379.

‡3. Genton, B.J., Jonot, O., Thévenet, D., Fournier, E., Blatrix, R., Vautrin, D. *et al.* 2005. Isolation of five polymorphic microsatellite loci in the invasive weed *Ambrosia artemisiifolia* (Asteraceae) using an enrichment protocol. *Mol. Ecol. Notes* **5:** 381-383.

**Appendix S2. Determination of microsatellite loci in (A) the population of potential parents and (B) the seed population (2102 seeds).**

Number of alleles, observed and expected heterozygosity and null allele frequency was calculated using INEST and Hardy-Weinberg test was made by CERVUS. HW represent the significance of deviation from Hardy-Weinberg equilibrium (*** = significant at the 0.1% level, ND = not done). The combined non-exclusion probability (second parent) was 2.87 × 10^-4^ in the population of potential parents, and 4.40 × 10^-4^ in the seed population. In the seed population, deviations from HW equilibrium were found in all loci.

(A)

| Locus | Number of alleles | Heterozygosity (Obs^1^) | Heterozygosity (Exp^2^) | HW | Null^3^ |
| --- | --- | --- | --- | --- | --- |
| *Amb82* | 22 | 0.579 | 0.919 | ND | 0.081 |
| *Ambart09* | 16 | 0.415 | 0.823 | *** | **0.151** |
| *Ambart21* | 14 | 0.695 | 0.888 | ND | 0.000 |
| *Ambart18* | 14 | 0.290 | 0.887 | ND | **0.298** |
| *Ambart06* | 20 | 0.800 | 0.930 | ND | 0.000 |
| *Ambart17* | 7 | 0.265 | 0.615 | *** | **0.329** |

(B)

| Locus | Number of alleles | Heterozygosity (Obs^1^) | Heterozygosity (Exp^2^) | HW | Null^3)^ |
| --- | --- | --- | --- | --- | --- |
| *Amb82* | 20 | 0.580 | 0.907 | *** | **0.100** |
| *Ambart09* | 17 | 0.259 | 0.770 | *** | **0.335** |
| *Ambart21* | 16 | 0.531 | 0.885 | *** | **0.128** |
| *Ambart18* | 19 | 0.387 | 0.868 | *** | **0.250** |
| *Ambart06* | 21 | 0.776 | 0.926 | *** | 0.000 |
| *Ambart17* | 7 | 0.226 | 0.584 | *** | **0.283** |

^1^ Obs represents the observed heterozygosity.

^2^ Exp represents the expected heterozygosity.

^3^ Bold letters indicate the null allele frequency was significantly larger than zero.

**Appendix S3. Effects of plant height and dry biomass on total or female reproductive investment in each population of *Ambrosia artemisiifolia*.** *P*-values were calculated after Bonferroni’s adjustment.

(A) Tsukuba City

|  | Effects on total reproductive investment | | | |  | Effects on total weight of seeds | | | |
| --- | --- | --- | --- | --- | --- | --- | --- | --- | --- |
|  | Estimate | SE | *F*_1,19_ | *P* |  | Estimate | SE | *F*_1,19_ | *P* |
| (Intercept) | 3.405 | 0.155 | - | - |  | 2.361 | 0.119 | - | - |
| Standardized height | 0.006 | 0.163 | 0.001 | > 0.05 |  | -0.141 | 0.125 | 1.269 | > 0.05 |
| Standardized dry biomass | 0.973 | 0.163 | 35.487 | < 0.001 |  | 0.899 | 0.125 | 51.834 | < 0.001 |

(B) Kouka City

|  | Effects on total reproductive investment | | | |  | Effects on total weight of seeds | | | |
| --- | --- | --- | --- | --- | --- | --- | --- | --- | --- |
|  | Estimate | SE | *F*_1,13_ | *P* |  | Estimate | SE | *F*_1,13_ | *P* |
| (Intercept) | 3.158 | 0.123 | - | - |  | 2.006 | 0.097 | - | - |
| Standardized height | 0.037 | 0.148 | 0.063 | > 0.05 |  | -0.012 | 0.117 | 0.011 | > 0.05 |
| Standardized dry biomass | 0.942 | 0.148 | 40.410 | < 0.001 |  | 0.811 | 0.117 | 48.183 | < 0.001 |

(C) Yamaguchi City

|  | Effects on total reproductive investment | | | |  | Effects on total weight of seeds | | | |
| --- | --- | --- | --- | --- | --- | --- | --- | --- | --- |
|  | Estimate | SE | *F*_1,14_ | *P* |  | Estimate | SE | *F*_1,14_ | *P* |
| (Intercept) | 3.182 | 0.143 | - | - |  | 2.105 | 0.136 | - | - |
| Standardized height | -0.215 | 0.160 | 1.804 | > 0.05 |  | -0.323 | 0.152 | 4.542 | > 0.05 |
| Standardized dry biomass | 0.371 | 0.160 | 5.366 | > 0.05 |  | 0.196 | 0.152 | 1.673 | > 0.05 |

**Appendix S4. Paternity determination**

We determined paternity for 1,306 out of 2,734 seeds (mean LOD score ± SD = 6.082 ± 2.954) by CERVUS.

| Combination | Number of seeds | | |
| --- | --- | --- | --- |
|  | Selfing | Outcrossing | Total |
| Northern mother – Northern father | 101 | 717 | 818 |
| Northern mother – Southern father | - | 333 | 333 |
| Southern mother – Northern father | - | 36 | 36 |
| Southern mother – Southern father | 20 | 99 | 119 |
| Total | 121 | 1185 | 1306 |

**Appendix S5. Measurements of 100 individuals used in the second experiment assessing the effects of plant size on fitness components.**

| Trait | Mean ± SD | Max | Min |
| --- | --- | --- | --- |
| Height (cm) | 77.61 ± 11.57 | 105.9 | 54.7 |
| Total raceme length (mm) | 2049.0 ± 1215.0 | 4835 | 0 |
| Number of seeds produced | 164.4 ± 173.1 | 862 | 0 |
| Total weight of seeds (g) | 0.640 ± 0.636 | 2.762 | 0 |
